# Construction of complete *Tupaia belangeri* transcriptome database by whole-genome and comprehensive RNA sequencing

**DOI:** 10.1038/s41598-019-48867-x

**Published:** 2019-08-26

**Authors:** Takahiro Sanada, Kyoko Tsukiyama-Kohara, Tadasu Shin-I, Naoki Yamamoto, Mohammad Enamul Hoque Kayesh, Daisuke Yamane, Jun-ichiro Takano, Yumiko Shiogama, Yasuhiro Yasutomi, Kazuho Ikeo, Takashi Gojobori, Masashi Mizokami, Michinori Kohara

**Affiliations:** 1 Department of Microbiology and Cell Biology, Tokyo Metropolitan Institute of Medical Science, 2-1-6, Kamikitazawa, Setagaya-ku, Tokyo 156-8506 Japan; 2 Transboundary Animal Diseases Centre, Joint Faculty of Veterinary Medicine, Kagoshima University, 1-21-24, Korimoto, Kagoshima, Kagoshima 890-0065 Japan; 3 Laboratory of Animal Hygiene, Joint Faculty of Veterinary Medicine, Kagoshima University, 1-21-24, Korimoto, Kagoshima, Kagoshima 890-0065 Japan; 4 BITS Co., Ltd., 1-5-5, Kandasurugadai, Chiyoda-ku, Tokyo 101-0062 Japan; 5 Department of Pathological and Preventive Veterinary Science, The United Graduate School of Veterinary Science, Yamaguchi University, 1677-1, Yoshida, Yamaguchi, Yamaguchi 753-8515 Japan; 6 Laboratory of Immunoregulation and Vaccine Research, Tsukuba Primate Research Center, National Institute of Biomedical Innovation, Health and Nutrition, 1-1 Hachimandai, Tsukuba, Ibaraki 305-0843 Japan; 7 National Institute of Genetics, 1111 Yata, Mishima, Shizuoka 411-8510 Japan; 8 King Abdullah University of Science and Technology, CBRC, 4700 KAUST, Thuwal, 23955-6900 Saudi Arabia; 9 Genome Medical Sciences Project, National Center for Global Health and Medicine, Ichikawa, Chiba 272-8516 Japan

**Keywords:** Genomic analysis, Genome

## Abstract

The northern tree shrew (*Tupaia belangeri*) possesses high potential as an animal model of human diseases and biology, given its genetic similarity to primates. Although genetic information on the tree shrew has already been published, some of the entire coding sequences (CDSs) of tree shrew genes remained incomplete, and the reliability of these CDSs remained difficult to determine. To improve the determination of tree shrew CDSs, we performed sequencing of the whole-genome, mRNA, and total RNA and integrated the resulting data. Additionally, we established criteria for the selection of reliable CDSs and annotated these sequences by comparison to the human transcriptome, resulting in the identification of complete CDSs for 12,612 tree shrew genes and yielding a more accurate tree shrew genome database (TupaiaBase: http://tupaiabase.org). Transcriptome profiles in hepatitis B virus infected tree shrew livers were analyzed for validation. Gene ontology analysis showed enriched transcriptional regulation at 1 day post-infection, namely in the “type I interferon signaling pathway”. Moreover, a negative regulator of type I interferon, *SOCS3*, was induced. This work, which provides a tree shrew CDS database based on genomic DNA and RNA sequencing, is expected to serve as a powerful tool for further development of the tree shrew model.

## Introduction

The northern tree shrew (*Tupaia belangeri*), which belongs to the family Tupaiidae, has a body weight ranging between 100–150 g, and is similar in appearance to squirrels^1^. The natural habitat of *Tupaia* spp. consists of the tropical rainforest in South East Asia where the animals feed on fruits, insects, and small vertebrates^1^. One of the appealing features of the tree shrew is its genetic closeness to human^2^. Thus, tree shrews are widely used as experimental animals in various research fields^3–6^. Recently, generation of a transgenic tree shrew has been reported^7^. The importance of tree shrew as an alternative animal model is growing.

Tree shrews have been employed in viral infection studies^8–10^, especially for hepatitis B virus (HBV)^11,12^ and hepatitis C virus (HCV)^13,14^, viruses for which the only other natural non-human infection model is chimpanzee. HBV, a member of the family *Hepadnaviridae*, causes acute and chronic hepatitis, and is a major worldwide public health concern^15^. Chronic HBV infection is strongly associated with an increased risk of cirrhosis, which in turn can lead to hepatocellular carcinoma^15,16^. Worldwide, 2 billion people have been reported to be infected with HBV, and more than 350 million have been reported to be chronically infected^15,17^. Urgent measures are required, but the development of drugs and vaccines has been hampered by the lack of an efficient animal model for infection by this virus. Since HBV causes human-like symptomology (including hepatitis and persistent infection) in tree shrews^11,12,18^, tree shrew could be a powerful animal model for HBV infection.

Genomic information is essential for various studies such as gene expression analysis and immunological analysis. Recently, an analysis of the tree shrew genome was reported^2,19^, and associated genomic information has been published in Ensembl (http://asia.ensembl.org) and the Tree shrew Database (TreeshrewDB: http://www.treeshrewdb.org). However, some of the entire coding sequences (CDSs) of tree shrew genes still remained incomplete. Additionally, even when CDSs have been identified, it can be difficult to determine how reliable those CDSs are. In the present study, we performed whole-genome sequencing, mRNA sequencing, and total RNA sequencing, and used the resulting data to select reliable complete tree shrew CDSs by utilizing defined criteria. Based on the obtained data, we have developed an enhanced tree shrew genome database (TupaiaBase: http://tupaiabase.org). In addition, we have validated this database using HBV-infected tree shrew liver specimens at an early stage of infection.

## Results

### Genome sequencing

In our initial work, we determined the whole-genome sequence of the tree shrew using next-generation sequencing. Starting from a tree shrew DNA sample, we constructed a series of libraries with different insert sizes (170 bp–20 Kb) (Table 1). Altogether, 14 libraries were generated, yielding a total of approximately 335 Gb of sequencing data. Assembly employed a subset of the data representing 240 Gb of high-quality sequence. The final N50 contig and total contig sizes were 33 Kb and 2.7 Gb, respectively (Table 2). The N50 scaffold and total scaffold sizes were 1.1 Mb and 2.7 Gb, respectively. As a next step, these sequencing data were annotated by homology-based gene prediction and *de novo* gene prediction, and a comprehensive gene set was constructed using GLEAN^20^. Based on GLEAN gene models, the tree shrew genome was predicted to contain a total of 19,320 protein-coding genes (Table 3).

**Table 1:** Statistics of whole-genome sequencing data.

Pair-end libraries	Insert size	Read length (bp)	Total Data (Gb)	Sequence depth (fold)*	Physical depth (fold)*
Illumina Reads	170 bp	100	60.96	19.82	16.84
	500 bp	100	72.67	23.63	59.06
	800 bp	100	51.25	16.66	66.65
	2 Kb	57	56.64	18.41	322.49
	5 Kb	49	35.94	11.68	596.03
	10 Kb	49	41.24	13.41	1368
	20 Kb	49	16.38	5.33	1087
Total			335.08	108.93	3516.07

^*^The genome size is assumed to be 3.08 Gb.

**Table 2:** Statistics of the assembled sequence length of whole-genome sequencing.

	Contig		Scaffold	
	Size (bp)	Number	Size (bp)	Number
N50	33,380	24,001	1,149,110	679
Longest	267,380	—	13,631,494	—
Total Size	2,709,670,168	—	2,746,321,810	—
Total number (>=100 bp)	—	585,492	—	447,618
Total number (>=2 kb)	—	121,063	—	7,608

**Table 3:** General statistics of predicted protein-coding genes of whole-genome sequencing.

Gene set	Number	Average gene length (bp)	Average CDS length (bp)	Average exon per gene	Average exon length (bp)	Average intron length (bp)
GLEAN	19,320	24,193	1,419	7.68	184.85	3,411

### Identification of CDSs by genome and RNA sequence analysis

To enhance the quality of gene annotation, HBV-infected tree shrews were used for RNA sequence (seq) analysis. Since an immune response should be induced under normal conditions, the use of HBV-infected tree shrew liver and spleen samples for RNA sequencing was expected to permit the identification of genes associated with an immune response to viral infection. In addition, different set of genes are expected to be expressed in each phase of infection. Therefore, RNA sequencing was performed on RNA samples obtained from HBV-infected tree shrew tissue samples at the acute phase of infection (at 1, 3, and 21 days post-infection (dpi)) and at the chronic phase of infection (at 8 months post-infection). In mRNA-seq analysis, over 200 million reads per group were sequenced, with over 90% of the bases of all samples exceeding Q30 (Table 4). Across all samples, 77 to 84% of the reads were mapped to the tree shrew genome. In total RNA-seq analysis, 88 to 126 million reads per sample were sequenced, with approximately 95% of the bases of all samples exceeding Q30. The percentages of total mapping reads of all samples were 86 to 91% (Table 5).

**Table 4:** mRNA-seq overview.

Sample	Total reads	% of >= Q30 bases	Trimmed reads	Total mapped reads	% of total mapped reads	Unmapped reads	% of unmapped reads
Uninfected tree shrew liver (#1)	51.46	91.12	49.70	40.02	80.53	9.68	19.47
Uninfected tree shrew liver (#2)	52.91	90.82	50.98	45.73	82.96	9.39	17.04
Uninfected tree shrew liver (#3)	57.20	90.99	55.12	40.86	80.15	10.12	19.85
Uninfected tree shrew liver (#4)	53.87	90.86	51.85	42.14	81.26	9.72	18.74
HBV-C 1 dpi tree shrew liver (#1)	94.47	90.76	91.16	76.55	83.97	14.61	16.03
HBV-C 1 dpi tree shrew liver (#2)	111.28	90.31	107.00	87.40	81.68	19.60	18.32
HBV-C 3 dpi tree shrew liver (#1)	93.80	90.60	90.49	72.73	80.37	17.76	19.63
HBV-C 3 dpi tree shrew liver (#2)	129.30	90.44	124.43	99.89	80.27	24.54	19.73
HBV-A 21 dpi tree shrew liver (#1)	200.98	90.15	191.47	149.06	77.85	42.41	22.15

Read values represent millions of reads.

**Table 5:** Total RNA-seq overview.

Sample	Total reads	% of >= Q30 bases	Trimmed reads	Total mapped reads	% of total mapped reads	Unmapped reads	% of unmapped reads
Uninfected tree shrew liver (#1)	117.38	94.87	115.39	100.37	86.99	15.02	13.01
Uninfected tree shrew liver (#2)	125.70	95.04	123.59	108.03	87.40	15.57	12.60
Uninfected tree shrew liver (#3)	92.43	94.74	90.28	82.01	90.84	8.27	9.16
Uninfected tree shrew liver (#4)	99.86	95.39	98.30	87.11	88.62	11.19	11.38
HBV-C 1 dpi tree shrew liver (#1)	106.45	95.33	104.75	92.07	87.90	12.68	12.10
HBV-C 1 dpi tree shrew liver (#2)	97.06	95.31	95.45	84.01	88.01	11.44	11.99
HBV-C 3 dpi tree shrew liver (#1)	99.51	95.51	97.87	85.30	87.15	12.57	12.85
HBV-C 3 dpi tree shrew liver (#2)	100.26	95.61	98.72	85.53	86.64	13.18	13.36
HBV-A 21 dpi tree shrew liver (#1)	94.93	95.57	93.48	80.42	86.02	13.07	13.98
Uninfected tree shrew spleen (mix; #1, #2, and #4)	91.34	95.43	89.69	79.00	88.09	10.69	11.91
HBV-C 1 and 3 dpi tree shrew spleen (mix; 1 dpi [#1, #2], 3 dpi [#2])	88.74	95.55	87.09	78.24	89.83	8.85	10.17
HBV-A 8 mpi tree shrew spleen (mix; #1, #2, and #3)	93.64	95.56	91.72	82.19	89.62	9.52	10.38

Read values represent millions of reads.

**Figure 1:**
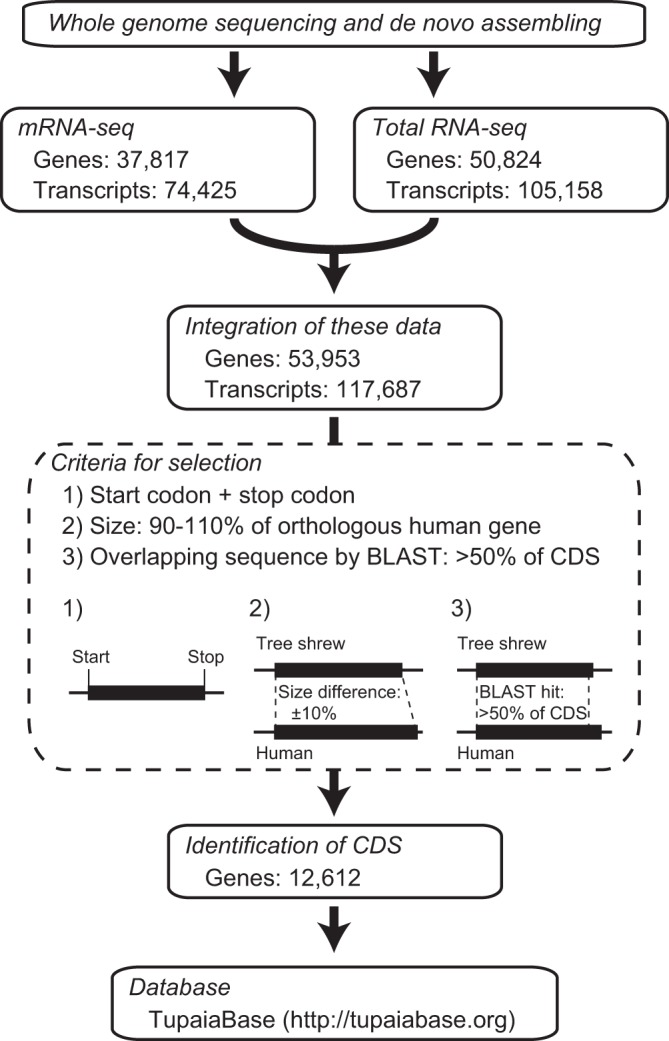
Schematic diagram of CDS identification of tree shrew genes.

A total of 74,425 transcripts and 37,817 genes were detected in the mRNA-seq analysis (Fig. 1). In the total RNA-seq analysis, a total of 105,158 transcripts and 50,824 genes were detected. The combination of these data yielded a total of 117,687 transcripts and 53,953 genes. We assessed the transcriptome assemblies using Benchmarking Universal Single-Copy Orthologs (BUSCO) software (Table 6). The number of total BUSCOs searched was 6,192. The number of complete BUSCOs in the combined transcriptome was 5,518 (89.1%), a value that exceeded the number (5,319; 85.9%) obtained using the previously available database results. Given the genetic similarity to human, these transcripts were annotated based on the Uniprot human protein database^21^. We then selected transcripts from each gene to identify the CDSs. To select only high-quality transcript data and determine the CDS of each gene, we set the following criteria for selection. (i) Start codon and stop codon existed in the transcript sequence. (ii) The lengths of the presumed gene product and the corresponding human orthologous gene product differed by less than 10%. (iii) The length of the overlap between the presumed tree shrew gene sequence and the corresponding human orthologous gene sequence (as aligned by BLASTX) constituted a distance of more than 50% of the presumed gene sequence. If there were multiple transcripts that met the above criteria for a given gene, the transcript that was most-strongly expressed was selected. Based on the criteria, we identified CDSs for a total of 12,612 genes.

**Table 6:** Evaluation of assemblies by BUSCO.

	TupaiaBase (Sanada *et al*. 2019)	TreeshrewDB (Fan *et al*. 2014)
Number of predicted genes	53,935	119,898
Number of predicted transcripts	117,687	192,459
BUSCO analysis		
Complete BUSCOs (%)	5,518 (89.1%)	5,319 (85.9%)
Fragmented BUSCOs (%)	398 (6.4%)	562 (8.1%)
Missing BUSCOs (%)	276 (4.5%)	311 (5.0%)
Total BUSCOs (%)	6,192 (100%)	6,192 (100%)

### Database construction

Using whole-genome sequencing, mRNA sequencing and total RNA sequencing data, we constructed a tree shrew genome database (TupaiaBase: http://tupaiabase.org). The JBrowse^22^ genome browser was used for the visualization of genome annotations.

### Gene sequence verification of database

**Figure 2:**
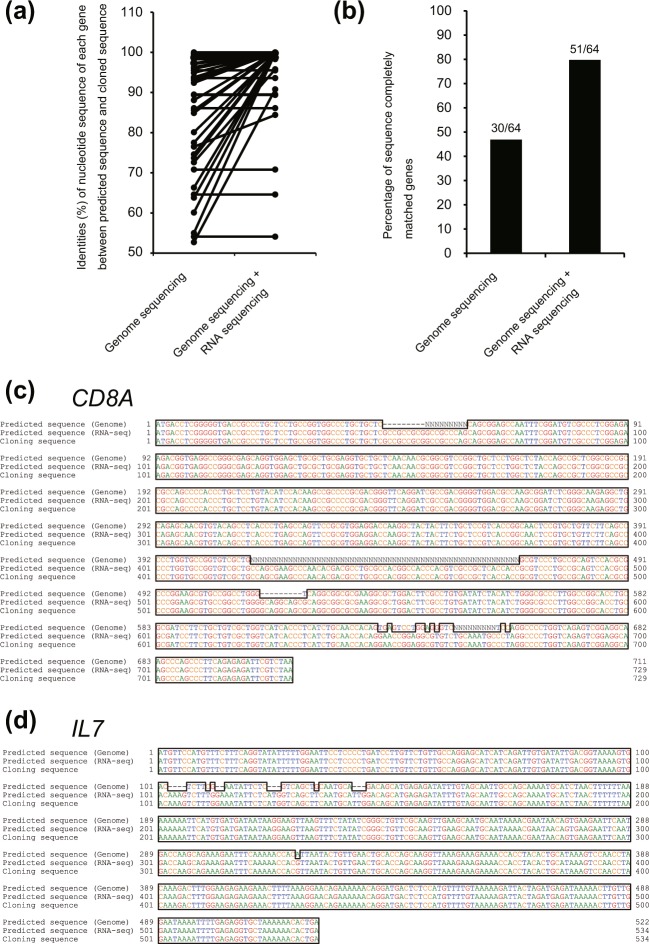
Analysis of the accuracy of the gene sequences predicted on the basis of the combined genome and RNA sequencing. (**a**) Identities of nucleotide sequences of each gene when comparing between the cloned sequence and the sequence predicted from whole-genome sequencing or between the cloned sequence and the sequence predicted from the combined sequence data from whole-genome sequencing and RNA sequencing (**b**) Percentage of sequence-completely-matched genes between the cloned sequence and sequence predicted from whole-genome sequencing or combined sequence data. (**c**,**d**) Comparison of predicted and actual gene sequences for *CD8A* (**c**) and *IL7* (**d**). Upper sequence: predicted sequence based on genome sequencing. Middle sequence: predicted sequence based on genome and RNA sequencing. Lower sequence: Cloned sequence.

To analyze the accuracy of the gene sequences predicted on the basis of the combined genome and RNA sequencing, we cloned a subset of genes, sequenced the resulting clones, and compared these sequences with the predicted versions. A total of 64 of these genes were successfully cloned and sequenced. Among the CDSs predicted solely on the basis of the genome sequence, 30 of 64 (46.9%) sequences were identical to the actual cloned sequence (Fig. 2a,b, and Supplementary Table S1); among the CDSs predicted by a combination of genome sequencing and RNA sequencing, 51 of 64 (79.7%) gene sequences were identical to actual cloned sequences. For example, the CDSs of the CD8 alpha (*CD8A*) and interleukin-7 (*IL7*) -encoding genes were not identified by genome sequencing alone, but these CDSs were detected by the combination of genome and RNA sequencing data. These predicted mRNA sequences did indeed match those of the actual cloned cDNA sequences (Fig. 2c,d). In a previous report, Yu *et al*. also determined the tree shrew *IL7* mRNA sequences^23^, and the CDS predicted by our method matched the canonical form of tree shrew *IL7* mRNA transcript (accession number: JQ182399). These results showed that gene sequence predictions based on the combination of genome and RNA sequencing were more accurate than those based on genome sequencing alone.

### Analysis of genes expressed in liver

To validate the 12,612 protein-coding genes identified in our analysis, we analyzed the expression of genes in liver. First, we determined how many genes were identified among genes expressed in liver. Among 426 genes categorized (in the human protein atlas: https://www.proteinatlas.org) as having “elevated expression in liver”, 366 genes were annotated in 1,766 transcripts from 117,687 transcripts not subjected to selection, and 274 genes (64.3%) with 290 transcripts from 12,612 selected transcripts were identified by our CDS selection criteria.

**Figure 3:**
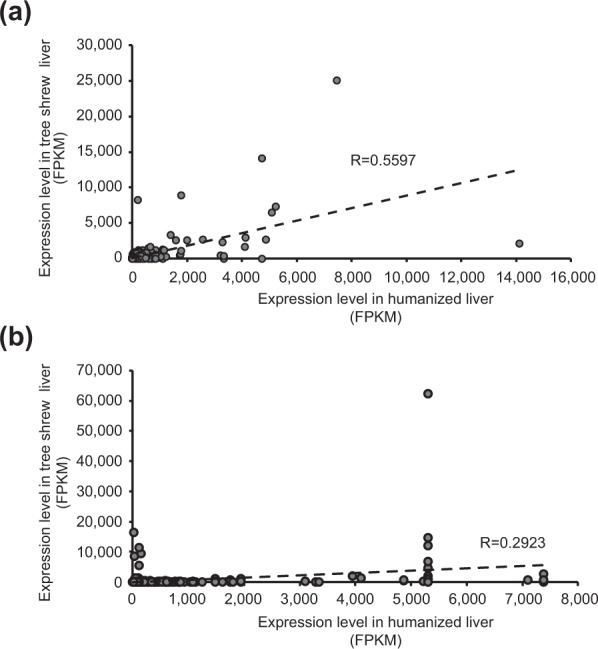
Expression level of liver-specific genes in tree shrew liver and in humanized liver in mouse. Correlation between gene expression level in humanized liver and homologous gene in tree shrew liver assessed for selected transcripts (**a**) or transcripts that failed to meet our criteria (**b**). Broken lines indicate regression curves.

Next, to evaluate the accuracy of the CDSs selected by our criteria, we compared the expression levels of 1,766 tree shrew transcripts with those of homologous human genes. Expression of functional transcripts in tree shrew was expected to correlate with the expression of the homologous human genes in liver. The genes expressed in human liver were analyzed using the livers of chimeric mice harboring humanized livers. The expression levels of tree shrew transcripts (290 in total) that met the criteria correlated well with the expression levels of transcripts of the homologous human genes (274 genes) (R = 0.5597) (Fig. 3a). On the other hand, the expression levels of tree shrew transcripts (1,476 transcripts) that failed to meet the criteria showed a poor correlation with the expression levels of transcripts of homologous human genes (R = 0.2923) (Fig. 3b). These data suggested that the identified CDSs selected by our criteria were more reliable than were excluded CDSs.

### Expression analysis of HBV-infected tree shrew

**Figure 4:**
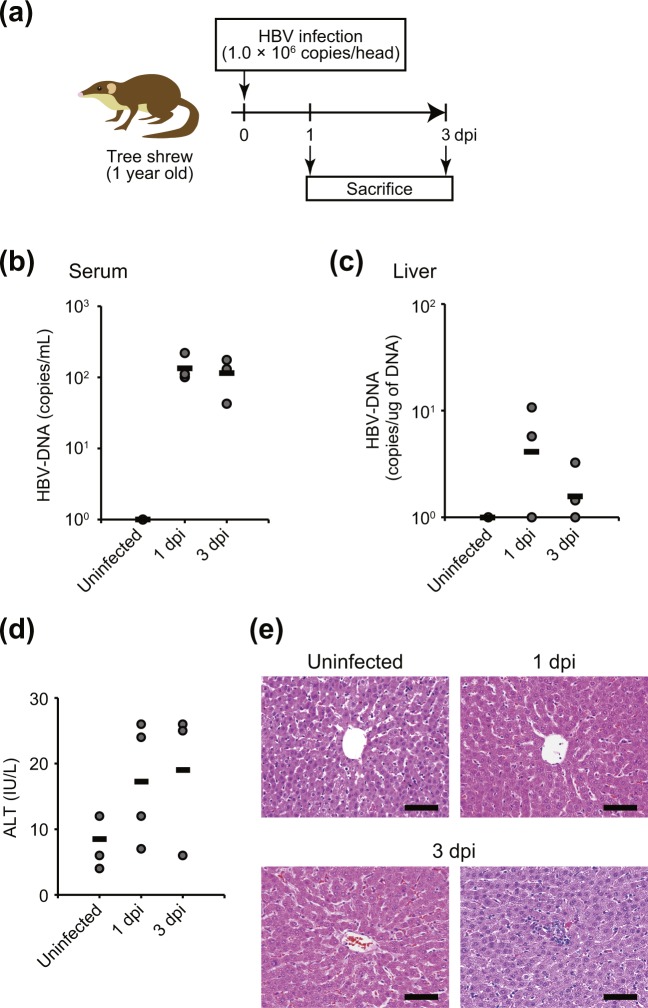
HBV infection in tree shrew. (**a**) Experimental schedule of HBV infection in tree shrew. (**b**–**d**) Viral DNA titers in sera (**b**) and liver (**c**), and serum ALT level (**d**), at 1 dpi or 3 dpi in HBV-infected tree shrew, or in uninfected tree shrew. Heavy bars indicate means of each group. (**e**) Histological analysis (hematoxylin-eosin staining; representative images) of liver from uninfected and HBV-infected (at 1 and 3 dpi) tree shrews. Bar, 100 μm.

It is difficult to identify accurately when HBV patients become initially infected; therefore, host response at the initial stage of HBV infection remains poorly documented. Hence, to have more insight in this regard, we analyzed the transcriptome profile of the identified genes in the early stage of HBV infection in tree shrew. We infected tree shrews by intravenous injection with HBV genotype C and sacrificed animals at 1 or 3 dpi (Fig. 4a). At 1 dpi, HBV viral loads were 1.0 × 10^2^ to 2.2 × 10^2^ copies/ml in serum samples, and 5.7 × 10^0^ to 1.1 × 10^1^ copies/μg liver DNA (Fig. 4b,c). At 3 dpi, HBV viral loads were 4.2 × 10^1^ to 1.8 × 10^2^ copies/ml in serum samples, and 1.4 × 10^0^ to 3.2 × 10^0^ copies/μg liver DNA. No viral DNA was detected from the livers of two tree shrews at 1 dpi, and from one tree shrew at 3 dpi. HBV infections caused nominal but non-significant elevation of serum ALT levels in some tree shrews (Fig. 4d). Interestingly, abnormal architecture of liver-cell cords was observed at 3 dpi, and lymphocytic infiltration also was observed by histochemical analysis (Fig. 4e).

**Figure 5:**
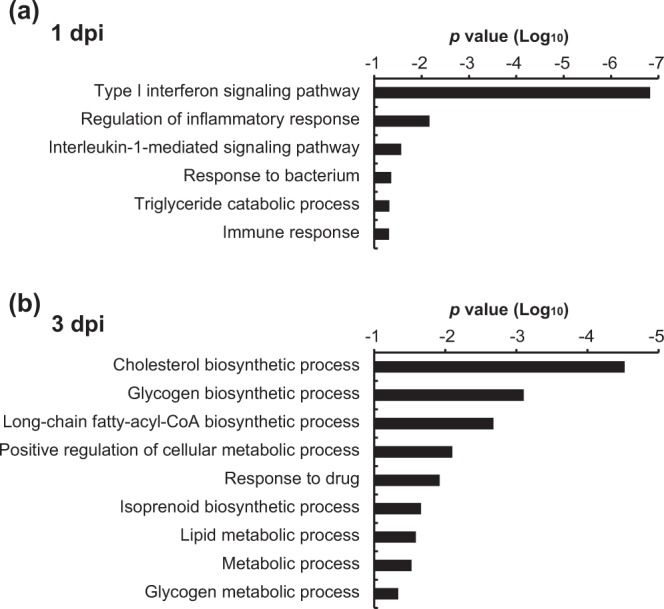
GO term analysis of differentially expressed genes in HBV-infected tree shrew at 1 dpi (**a**) and 3 dpi (**b**).

Transcriptome analysis showed that the number of differentially expressed genes (DEGs) at 1 and 3 dpi were 35 and 28, respectively. To characterize the DEGs, we performed GO term analysis of the DEGs at each time point (Fig. 5). The GO term characteristics of DEGs at 1 and 3 dpi were distinct. At 1 dpi, the GO terms of DEGs were primarily immune response-related (e.g., “Type I interferon signaling pathway” and “Regulation of inflammatory response”) (Fig. 5a). At 3 dpi, the GO terms of DEGs were mainly related to “Cholesterol biosynthetic process” and “Glycogen biosynthetic process” (Fig. 5b). These results implied that the primary immune response of tree shrew to HBV infection was attenuated rapidly (within 3 days).

### Analysis of genes related to type I interferon signaling

**Figure 6:**
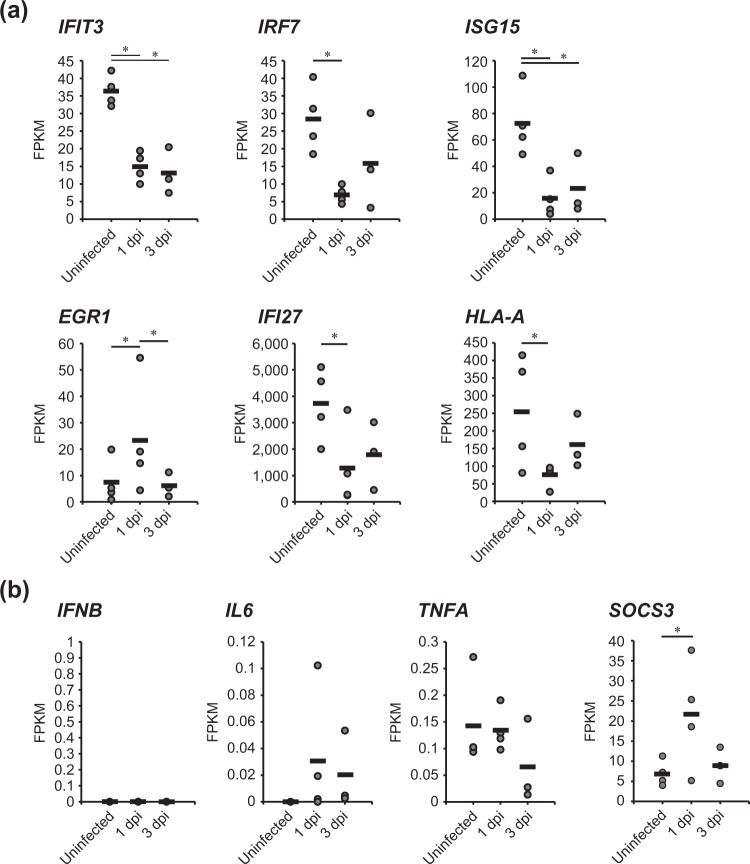
Expression analysis of genes related to type I interferon signaling. (**a**) Expression levels (in uninfected tree shrew and in HBV-infected tree shrew at 1 and 3 dpi) of genes whose GO included the term “type I interferon signaling pathway” and exhibited the strongest differential expression genes at 1 dpi. (**b**) Expression levels (in uninfected tree shrew and in HBV-infected tree shrew at 1 and 3 dpi) of genes known to be central to the type I interferon signaling pathway. Horizontal bars indicate mean values in each group. Asterisks indicate significant differences (*p* < 0.05).

Since GO term analysis revealed expression changes at 1 dpi in genes related to the type I interferon signaling pathway, we analyzed the expression level of DEGs categorized as part of the “type I interferon signaling pathway”. A total of 6 DEGs related to the type I interferon signaling pathway were observed at 1 dpi (Fig. 6a). The gene designations (using the names of the orthologous human genes) were as follows: interferon-induced protein with tetratricopeptide repeats 3 (*IFIT3*), interferon regulatory factor 7 (*IRF7*), ubiquitin-like protein interferon-stimulated gene 15 (*ISG15*), early growth response protein 1 (*EGR1*), interferon alpha-inducible protein 27 (*IFI27*), and HLA class I histocompatibility antigen, A-69 alpha chain (*HLA-A*). Notably, following viral infection, the levels of these transcripts (with the exception of *EGR1*) decreased at 1 dpi, with the levels of *IFIT3* and *ISG15* remaining significantly depleted at 3 dpi.

Our previous study showed that HBV infection suppressed or did not induce the expression of the interferon beta-encoding gene (*IFNB*) in tree shrew at 4 and 31 weeks post-infection^24^. To clarify whether early-stage HBV infection induced the expression of genes that are key factors in the type I interferon signaling pathway, we analyzed the mRNA expression levels of *IFNB* and the genes encoding interleukin-6 (*IL6*), and tumor necrosis factor alpha (*TNFA*). Notably, expression of these genes did not differ significantly when comparing between uninfected and HBV-infected (at 1 and 3 dpi) tree shrews (Fig. 6b). A recent study showed that the HBx protein suppresses type I interferon signaling by upregulating expression of the suppressor of cytokine signaling 3-encoding gene (*SOCS3*)^25^. Indeed, in HBV-infected tree shrew, expression of the *SOCS3* transcript was significantly upregulated at 1 dpi (Fig. 6b). These data suggested that the initial phase of HBV infection in tree shrew induces the expression of the *SOCS3* gene, resulting in suppression of the type I interferon response.

## Discussion

Recently, tree shrew has been widely used for various biological studies, including investigations of viral infection^11,12,14^, depression^3^, the visual system^4^, and so on. However, research tools (e.g., antibody, PCR system) are limited for the establishment of tree shrew as an experimental animal model. Although these tools are based on genomic information, some of the entire CDSs of genes still had not been completed. Even in cases for which the CDS had been determined, it remained difficult to determine how reliable that CDS was. In the present study, we combined whole-genome analysis with RNA sequencing data, and selected 12,612 genes for which the CDS seemed to be accurate. We then published these tree shrew genomic data as part of a publicly available tree shrew database.

Based on whole-genome analysis alone, it is difficult to predict the entire CDSs of a genome. Comparing the predicted sequences based on whole-genome analysis with actual cDNA sequences revealed that approximately half of the cloned sequences did not match the predicted sequences. This observation reflects, in part, how difficult it is to determine the sequences of long genes and those of genes that contain short exons. To improve the accuracy of predicted CDSs, we combined whole-genome analysis and RNA sequencing data. RNA sequencing reads were mapped to the tree shrew genome, enabling identification of the CDSs of individual genes. About 80% of the cDNA sequences predicted on the basis of the combination of whole-genome sequencing and RNA sequencing were identical to actual cloned gene sequences; the remaining 20% of the predicted cDNA sequences were not recovered as actual cDNA sequences, presumably reflecting the low level of expression of these genes under the conditions of our experiment. Since gene expression levels may differ in various organs and under other conditions, experiments to sequence the RNAs collected from other organs and under other conditions will be needed.

In the present study, we focused on tree shrew genes sharing high homology with orthologous human genes, and determined the CDSs of 12,612 genes. To validate the protein-coding genes identified in our analysis, we analyzed the expression of transcripts annotated as liver-specific genes. Expression of selected transcripts (i.e., those that met the stated criteria) in tree shrew liver showed better correlation with that of homologous human genes in humanized liver (R = 0.5597) than with that of excluded transcripts (i.e., those that did not meet these criteria) (R = 0.2923). These data suggested that CDSs selected by our criteria are more reliable than unselected CDSs. Of course, our selection is far from being perfect, given that we focused on the tree shrew genes sharing high sequence similarity with the corresponding human genes. We are currently performing an optimization of the criteria for transcript selection, with the expectation that this analysis will permit identification of tree shrew-specific genes.

In the present study, we constructed a tree shrew genome database (TupaiaBase: http://tupaiabase.org). The first feature of our database is the quality of data. Complete BUSCOs of this study is 89.1%; thus, our database is thought to be based on high-quality data. The second feature of our database is that the database uses JBrowse for the visualization of genome annotations. JBrowse has been used extensively in other databases, including Mouse Genome Informatics (MGI; http://www.informatics.jax.org), Rat Genome Database (RGD; https://rgd.mcw.edu), and FlyBase (https://flybase.org), and is known to be fast and easy to use. The third feature of our database is the availability of the CDSs. Cloned sequences and selected genes in this study are displayed, permitting the user to readily identify reliable CDSs. Though a tree shrew database (TreeshrewDB) is already available, these features of our tree shrew genome database (TupaiaBase) are expected to help facilitate advances in research in this organism. The utility of our database should extend the potential of tree shrew as an animal model in various research fields.

In practice, it is difficult to identify when HBV patients become initially infected, and thus the collection of samples from humans at the initial stage of HBV infection has not been possible. This shortcoming has precluded the determination of the host response in the early stages of HBV infection in humans. Tree shrew infected with HBV exhibits hepatitis that resembles human cases, and therefore is expected to serve as a useful tool for clarifying the host response to HBV infection. Indeed, by using the tree shrew model, we were able to perform a preliminary analysis of the dynamics of transcript accumulation in the initial phase of HBV infection. Kouwaki *et al*. also have shown that early-stage HBV infection induces hepatic interferon gamma expression in tree shrew^26^. These results indicate that the tree shrew HBV infection model will be of great value in clarifying the early *in vivo* response to HBV infection in animals with intact immune systems.

The present study revealed that the expression of tree shrew genes related to the type I interferon signaling pathway is downregulated at 1 dpi. Our study also showed that genes encoding key factors (e.g., *IFNB*) known to be involved in the type I interferon response were not induced in the earliest stage of HBV infection of tree shrew. However, upregulation of *SOCS3* at 1 dpi was observed in our study, an observation consistent with a recent report that *SOCS3* is induced by HBx protein in human cell culture, thereby suppressing the type I interferon response^25^. Although the upregulation of *SOCS3* expression has been detected in the liver of chronically HBV-infected patients^25,27^, the role and kinetics of *SOCS3* expression during the initial phase of HBV infection *in vivo* remains unknown. Our study suggests that upregulation of *SOCS3* may facilitate the initial propagation of HBV *in vivo*. Further studies using the tree shrew model are expected to elucidate the role of *SOCS3* in HBV infection and to reveal other key factors involved in the initial phases of HBV infection.

In conclusion, we have constructed a database that identifies tree shrew CDSs based on a combination of genome and RNA sequencing. To improve this database, we are planning to perform long-read sequencing to enhance recovery of the complete genome with the correct order; we will continue to incorporate the new sequencing data as part of this database. Since tree shrew is increasingly being used in various research fields, this database is expected to serve as a powerful tool for further development of the tree shrew model and thus for enhancement of associated research.

## Methods

### Ethics statement

This study was carried out in strict accordance with the *Guidelines for Animal Experimentation of the Japanese Association for Laboratory Animal Science* and the recommendations in the *Guide for the Care and Use of Laboratory Animals* of the National Institutes of Health. All protocols were approved by the Committee on the Ethics of Animal Experiments of the Tokyo Metropolitan Institute of Medical Science.

### Animals

Northern tree shrews (*T*. *belangeri*) were purchased from the Kunming Institute of Zoology, Chinese Academy of Sciences. The animals were bred at Kagoshima University and the Tsukuba Primate Research Center for further experimental use.

Chimeric mice with humanized livers were purchased from PhoenixBio (Hiroshima, Japan).

### Genome sequencing

A male four-year-old tree shrew was used for whole-genome sequencing. The whole-genome sequencing of tree shrew was performed by BGI (Shenzhen, China). Using a tree shrew DNA sample, libraries of different insert sizes (170 bp, 500 bp, 800 bp, 2 Kb, 5 Kb, 10 Kb, and 20 Kb) were constructed by standard Illumina library preparation protocols. The tree shrew genome was assembled from massive reads using software in the SOAPdenovo (Short Oligonucleotide Assembly Program; version 1.05)^28^ package by the following steps. First, sequencing errors on raw reads were corrected based on K-mer frequencies using KmerFreq and Corrector. Then, contigs and scaffolds were built using the SOAPdenovo assembler. Finally, gaps were closed using GapCloser. To predict genes in the tree shrew genome, homology-based gene prediction and *de novo* gene prediction were performed. For the homology-based gene prediction, human (*Homo sapiens*), gorilla (*Gorilla gorilla*), rhesus macaque (*Macaca mulatta*), lemur (*Microcebus murinus*), and gibbon (*Nomascus leucogenys*) proteins were mapped onto the tree shrew genome to define the putative genes. For *de novo* gene prediction, two *de novo* prediction programs (GENSCAN^29^ and Augustus^30^ (version 2.5.5)) were used. Using GLEAN^20^ (version 1.0.1), these gene sets were combined to yield a comprehensive gene set.

### HBV inoculum

The HBV inoculum was the serum of a chimeric mouse with humanized liver that had been infected with HBV genotype A2 and C (C_JPNAT; accession number: AB246345.1).

### HBV infection of animals

One-year-old tree shrews (n = 11) were inoculated intravenously with 1.0 × 10^7^ viral DNA copies of HBV. Animals were sacrificed at 1 dpi (n = 4), 3 dpi (n = 3), 21 dpi (n = 1), and 8 months post-infection (mpi) (n = 3). Animals at 1 and 3 dpi were used for RNA sequencing and transcriptome analysis. Animals at 21 dpi and 8 mpi were used only for RNA sequencing.

### RNA extraction and sequencing

Total RNA was isolated from liver and spleen tissues from uninfected (n = 4) and HBV-infected tree shrews at 1 dpi (n = 4), 3 dpi (n = 3), 21 dpi (n = 1), and 8 mpi (n = 3). Total RNA was purified using the AGPC and RNeasy kit (Qiagen, Hilden, Germany). For mRNA sequencing, the mRNA in each total RNA sample was converted into a cDNA library using the TruSeq Standard mRNA prep kit (Illumina, San Diego, CA, United States). For total RNA sequencing, each RNA sample was converted into a cDNA library using the TruSeq Standard Total RNA prep kit (Illumina). These libraries then were sequenced using a HiSeq2000 sequencer (Ilumina). All reads were mapped to the tree shrew genome using TopHat (version 2.0.10)^31^ with an option that enabled the identification of micro-exons (–microexon-search). Transcripts were assembled and abundances were estimated using Cufflinks (version 2.1.1)^32^. The data from the mRNA sequencing and total RNA sequencing were merged using Cuffmerge, an application that is included as part of the Cufflinks package. Transcripts were annotated by comparison to human Uniprot protein data using BLAST+ (version 2.2.29+)^33^.

Total RNA from the livers of uninfected chimeric mice with humanized livers (n = 3) was isolated, purified, and used for mRNA sequencing as described above. Reads were mapped to the human reference genome (GRCh37 release 75) using TopHat (version 2.0.14). Transcripts were assembled and abundances were estimated using Cufflinks (version 2.2.1).

### Comparison with publicly available data

Publicly available RNA sequencing data for the Northern tree shrew were obtained from NCBI SRA listed in TreeshrewDB. The accession numbers of the data obtained were as follows: SRX1009946, SRX1017387, SRX125163, SRX157960, SRX157961, SRX157962, SRX157963, SRX157964, SRX157965, SRX157966, SRX3341772, SRX3358315, SRX3358316, SRX3358317, SRX3358318, and SRX3358319. These data were analyzed by the same informatics process as described above.

The completeness of transcript sets of our data and publicly available data were assessed using BUSCO software^34^ (version 3.0.2).

### Identification of gene CDSs

To select only high-quality transcript data and determine the CDS of each gene, we used the following criteria for selection. (i) Both a start codon and a stop codon were present in the transcript sequence. (ii) The lengths of the presumed gene product and the corresponding orthologous human gene product differed by less than 10%. (iii) The length of the overlap between the presumed tree shrew gene sequence and the corresponding orthologous human gene sequence (as aligned by BLASTX with an option that set the maximum expectation value to 10^−5^) constituted a length of more than 50% of the presumed gene sequence. If there were multiple transcripts that met the above criteria for a given gene, the transcript showing highest expression was selected.

### Prediction of CDSs by genome sequencing

To predict the CDSs of genes by genome sequencing, the tree shrew genome sequencing data were searched for gene sequences by BLASTX with human gene sequences. Since exons of each gene were separated, sequences that showed overlap by BLASTX were connected and considered as a single predicted CDS in the genome sequence.

### Cloning of genes

To perform the cloning of genes, we predicted both the 5′- and 3′ -end sequences of each gene CDS. Sequences at both ends were predicted from the genome sequence as described above. Gene cloning was performed by Takara Bio (Shiga, Japan). The target sequences were reverse transcribed and amplified by PCR. The resulting PCR fragments were cloned into the pCAGGS vector.

### Analysis of gene expression

To analyze the mRNA expression level in tree shrew liver, FPKM (fragments per Kb of exon model per million mapped fragments) values were used for normalization. Differentially expressed genes were categorized by gene ontology (GO)^35^ and analyzed by enriched GO using DAVID software (version 6.8)^36^.

### HBV-DNA quantification

Viral DNA was extracted using SMItest EX-R&D kits (Nippon Genetics, Tokyo, Japan) according to the manufacturer’s instructions. Quantification of HBV-DNA was performed using real-time detection PCR, as previously described^37^. The primers and probes for the S gene consisted of forward primer HB-166-S21 (nucleotides (nts) 166–186: 5′-CACATCAGGATTCCTAGGACC-3′), reverse primer HB-344-R20 (nts 344–325: 5′-AGGTTGGTGAGTGATTGGAG-3′), and TaqMan probe HB-242-S26FT (nts 242–267: 5′-CAGAGTCTACACTCGTGGTGGACTTC-3′).

### Measurement of serum alanine aminotransferase (ALT) activity

Serum ALT activity in tree shrew was determined using the Transaminase CII-test Wako (Wako Pure Chemical Industries, Osaka, Japan) according to the manufacturer’s instructions.

### Histological analysis

Tree shrew liver tissues were fixed with 10% phosphate-buffered formalin and embedded in paraffin. The samples then were sectioned and stained with hematoxylin and eosin using standard methodologies.

### Statistical analyses

*p* values lower than 0.05 were considered significant. Cuffdiff, included in Cufflinks, was used to estimate the statistical significance of gene expression changes between sample groups.

### Accession numbers

The raw data of the whole-genome sequencing and RNA sequencing have been deposited in the DNA Data Bank of Japan (DDBJ) with accession numbers DRR155071-DRR155099.

## Supplementary information

Dataset 1

## Data Availability

All data that support the findings of this study are available from the corresponding authors upon reasonable request.
